# Evaluation of GDF15 Significance as a Biomarker in Laryngeal Squamous Cell Carcinoma

**DOI:** 10.3390/jcm14144870

**Published:** 2025-07-09

**Authors:** Aleksandra Romanowicz, Oskar Komisarek, Anna Klimaszewska-Wiśniewska, Paulina Antosik, Kacper Naglik, Joanna Czech, Witold Wrzesiński, Marta Kodzik, Magdalena Bodnar, Dariusz Grzanka, Paweł Burduk

**Affiliations:** 1 Department of Otolaryngology, Laryngological Oncology and Maxillofacial Surgery, University Hospital, 85-168 Bydgoszcz, Poland; romanowicz.aleks@gmail.com (A.R.); otolaryngologia@biziel.pl (J.C.); wiwrz@tlen.pl (W.W.); kodzik@poczta.onet.eu (M.K.); pburduk@wp.pl (P.B.); 2 Department of Otolaryngology, Phoniatrics and Audiology, Faculty of Medicine, Collegium Medicum, Nicolaus Copernicus University in Toruń, 87-100 Toruń, Poland; 3 Department of Clinical Pathomorphology, Collegium Medicum in Bydgoszcz, Nicolaus Copernicus University in Toruń, 87-100 Toruń, Poland; ania.klimaszewska@op.pl (A.K.-W.); paulina.antosik@cm.umk.pl (P.A.); kapi.n@wp.pl (K.N.); d_grzanka@cm.umk.pl (D.G.); 4 Department of Obstetrics, Women’s Diseases and Gynaecological Oncology, Collegium Medicum in Bydgoszcz, Nicolaus Copernicus University in Toruń, 87-100 Toruń, Poland; magdalena.bodnar@cm.umk.pl

**Keywords:** laryngeal squamous cell carcinoma, GDF15, tissue macroarrays, immunohistochemistry

## Abstract

**Background/Objectives**: Laryngeal squamous cell carcinoma (LSCC) is a common malignancy with unsatisfactory survival rates, highlighting the need for reliable biomarkers to improve its diagnosis and prognosis. Growth differentiation factor 15 (GDF15), a protein implicated in various cancers, has not been thoroughly investigated in LSCC. This study aimed to evaluate the significance of GDF15 expression in LSCC by integrating immunohistochemical analysis of archival tissue samples with RNA sequencing data from public databases (TCGA and GEO) to provide comprehensive clinical insights. **Methods**: We analyzed archival tissue samples from 65 patients with LSCC using immunohistochemistry and evaluated GDF15 expression profiles from The Cancer Genome Atlas (TCGA) and Gene Expression Omnibus (GEO) datasets. Statistical analyses included Kaplan–Meier survival analysis and Cox proportional hazards regression to assess the correlation between GDF15 expression, clinicopathological variables, and survival outcomes. **Results**: GDF15 expression did not significantly differ between tumor and adjacent normal tissues. However, in the tissue macroarray (TMA) cohort, high GDF15 expression was significantly associated with a lower TNM stage and less advanced pT status. Kaplan–Meier analysis revealed that high GDF15 expression correlated with reduced overall survival in the TMA cohort, suggesting its utility in risk stratification. Multivariate analysis identified GDF15 as an independent prognostic factor for disease-free survival in LSCC. **Conclusions**: Our findings suggest that GDF15 may serve as a prognostic biomarker for LSCC, particularly in early-stage disease. Elevated GDF15 levels, which are associated with poorer overall survival, could be integrated into diagnostic panels to enhance risk stratification and inform treatment decisions. Furthermore, GDF15 may be a promising target for therapeutic intervention. Further research is warranted to validate these results and explore their potential in clinical practice.

## Simple Summary

Laryngeal squamous cell carcinoma (LSCC) is a common head and neck cancer with limited survival rates despite advances in treatment options. Identifying reliable biomarkers could improve early diagnosis and treatment outcomes. In this study, we investigated the growth differentiation factor 15 (GDF15), a protein with roles in various cancers, to determine its significance in LSCC. By analyzing tissue samples from patients and public datasets, we assessed whether GDF15 expression correlated with clinical factors and survival outcomes. Our findings suggest that GDF15 may serve as a prognostic biomarker for LSCC, potentially aiding in patient stratification and treatment planning. This research contributes to a better understanding of GDF15’s role in cancer and highlights its potential utility in improving LSCC management. Future studies could build on these insights to explore GDF15’s role as a therapeutic target.

## 1. Introduction

Laryngeal carcinoma is the second most common malignancy of the aerodigestive tract, comprising approximately 20% of head and neck cancers. It occurs nearly five times more frequently in men than in women. The vast majority of cases are squamous cell carcinomas (LSCC), with tobacco and alcohol consumption being the most significant risk factors [1,2,3,4,5]. Patients are frequently diagnosed at an advanced stage, and the 5-year survival rate remains around 65% [3]. Despite the availability of radiotherapy and systemic treatments, surgery continues to be a cornerstone of management, depending on the disease stage and local extension.

Due to the still unsatisfactory prognosis, there is a pressing need to identify novel and reliable biomarkers to improve risk stratification and guide therapeutic decisions. Currently used biomarkers, such as p16, EGFR, and HPV status, often lack sensitivity and specificity. Growth differentiation factor 15 (GDF15), a divergent member of the TGF-β superfamily, has emerged as a potential candidate biomarker because of its involvement in inflammation, cellular stress response, and tumor progression [6,7,8].

GDF15, also known as MIC-1, PLAB, or PDF, is encoded on chromosome 19p12–13 and plays a pleiotropic role in multiple tissues, including the placenta, liver, kidney, and macrophages. It exerts both pro- and anti-tumorigenic effects, depending on the cancer type and tumor microenvironment [8,9]. Mechanistically, GDF15 engages multiple signaling pathways, including ERK, PI3K/AKT, and MAPK, and is a transcriptional target of p53 [8]. Overexpression of this gene has been observed in several cancers, including breast, cervix, prostate, colon, and oral cavity cancers, and is often associated with poor prognosis [10,11,12,13,14,15,16,17,18,19].

However, the role of GDF15 in laryngeal squamous cell carcinoma remains unexplored. This study aimed to comprehensively evaluate the clinical significance of this association in LSCC. By integrating immunohistochemical analyses of tumor tissue with transcriptomic data from TCGA and GEO, we aimed to assess the diagnostic and prognostic potential of GDF15 in this type of cancer. The rationale for selecting GDF15 stems from its emerging role in tumor progression, immune modulation, and cancer-related systemic effects such as cachexia. While GDF15 has been implicated in various malignancies, including prostate, pancreatic, and gastric cancers, its role in LSCC remains unexplored. Our study is the first to systematically evaluate GDF15 expression in LSCC using both protein and mRNA-level analyses, addressing a significant knowledge gap in biomarker research for this cancer type.

## 2. Materials and Methods

### 2.1. Patients and Tissue Material

The study was conducted using archival formalin-fixed, paraffin-embedded (FFPE) tissue samples obtained between 2016 and 2020 from patients diagnosed with laryngeal squamous cell carcinoma (LSCC) at the Department of Otolaryngology, Oncological Laryngology, and Maxillofacial Surgery, University Hospital No. 2, Bydgoszcz, and the Department of Clinical Pathomorphology, Collegium Medicum in Bydgoszcz, Nicolaus Copernicus University, Toruń, Poland. The diagnosis was confirmed by two independent pathologists using hematoxylin and eosin (H&E)-stained sections.

A total of 85 patients were initially screened. Twenty patients were excluded: 10 due to prior oncologic treatment, 5 due to the presence of carcinoma in situ or a second primary tumor, and 5 due to the absence of tumor cells on immunohistochemical staining. This resulted in a final study cohort of 65 patients, referred to as the tissue macroarray (TMA) cohort.

Tumor samples were reclassified according to the AJCC 8th edition TNM criteria to ensure consistency. Adjacent non-tumorous tissues (NATs) were collected from all surgical specimens; however, only 21 NAT samples met the predefined quality thresholds (≥70% epithelial cellularity and preserved morphology). Samples that failed to meet these criteria were excluded due to tissue degradation, fragmentation, or insufficient epithelial content. All NATs included in the final analysis were histologically confirmed to be free of malignant cells.

The clinicopathological data of the 65 patients are presented in Table 1. The study protocol was approved by the Bioethics Committee of Collegium Medicum in Bydgoszcz of Nicolaus Copernicus University in Toruń (KB 58/2022) and was conducted in accordance with the Declaration of Helsinki.

### 2.2. Survival Definitions

Overall survival (OS) was defined as the time from the date of curative surgery to death from any cause. Disease-free survival (DFS) was defined as the time from the date of curative surgery to the time of relapse or death. OS and DFS data were censored for patients who were alive at the last follow-up (11 January 2023). The median follow-up time, calculated using the reverse Kaplan–Meier method, was 49 months for OS and 46 months for DFS.

### 2.3. Tissue Macroarray Construction and Immunohistochemistry

Formalin-fixed, paraffin-embedded (FFPE) tissue samples were used to construct tissue macroarrays (TMA). Representative tumor areas with ≥80% tumor cell content were identified on hematoxylin and eosin (H&E) stained slides. Tissue cores were extracted and inserted into recipient blocks, which were sectioned at 3 µm using a rotary microtome and placed on high-adhesive, glass slides.

Immunohistochemical (IHC) staining was performed using an automated BenchMark^®^ Ultra platform (Roche Diagnostics/Ventana Medical Systems, Tucson, AZ, USA) and the UltraView DAB detection system. The sections were incubated with a rabbit polyclonal anti-GDF15 antibody (HPA011191, Sigma-Aldrich, St. Louis, MO, USA) at 1:150 dilution for 32 min. Positive and negative controls were also included.

Two independent pathologists evaluated GDF15 protein expression using a modified immunoreactive score (IRS), which was calculated by multiplying the staining intensity (0–3) by the percentage of positive cells (0–4). The average of three randomly selected fields at 20× magnification was used. Expression was dichotomized into low (IRS < 4) and high (IRS ≥ 4) groups based on the Evaluate Cutpoints software.

### 2.4. Extraction of RNA Sequencing TCGA Data and Clinical Information

RNA sequencing data (DESeq2 normalized counts) for GDF15 expression in 116 LSCC tissues and 12 adjacent tissues were downloaded from The Cancer Genome Atlas (TCGA) using the University of California Santa Cruz (UCSC) Xena Browser (https://xenabrowser.net/, accessed on 10 February 2023), whereas corresponding clinical information was retrieved from cBioPortal (https://www.cbioportal.org, accessed on 10 February 2023). The clinical endpoints were OS and DFS. The former was defined as the time from the date of curative surgery to the time of death from any cause, while the latter was defined as the time from the date of curative surgery to the time of recurrence or progression. Overall survival and DFS data were available for 116 (100%) and 87 (75%) patients. GDF15 expression levels were categorized into low- and high-level groups based on the optimal cut-off values determined using the Evaluate Cutpoints software [20]. The cut-off values for OS and DFS were 9.731 (<9.731, low expression; ≥9.731, high expression) and 7.217 (<7.217, low expression; ≥7.217, high expression), respectively. The median OS and DFS in the TCGA cohort were 65 and 76 months, respectively. Due to methodological and technical differences between datasets, different cut-off values were applied to define high versus low GDF15 expression in each cohort. In the TMA cohort, protein expression was dichotomized using Evaluate Cutpoints software based on IRS values to maximize prognostic discrimination. In the TCGA cohort, two separate optimal thresholds were generated using the same software: 9.731 for overall survival (OS) and 7.217 for disease-free survival (DFS). These values reflect differences in the expression distribution and survival-specific risk stratification.

In the GEO microarray datasets, which contained normalized gene expression values with distinct dynamic ranges, median expression values were used as cut-offs for dichotomization. Although this introduces variability, each cut-off was statistically validated within its respective dataset. As the datasets differ in platform (IHC vs. RNA-Seq vs. microarray) and data structure, internal validation was deemed most appropriate to preserve prognostic value while avoiding artificial harmonization across biologically distinct data types.

### 2.5. GEO Data Collection

The expression levels of GDF15 were extracted from the Gene Expression Omnibus (GEO) database through the ShinyGEO web-based application (http://gdancik.github.io/shinyGEO/, accessed on 13 March 2023) [20]. Five GEO datasets for LSCC: GSE25727 [21], GSE27020 [22], GSE59102 [23], GSE117973 [24,25], and GSE51985 [26] were included. If several probes were mapped to GDF15, the mean value was used as the final expression of this gene. In addition, GEO data were used as an independent validation of our GDF15 expression results. Due to the limited number of clinical samples available in our study (65 LSCC cases and only 21 NAT samples), integrating publicly available GEO datasets allowed us to extend our analysis and compare our findings with those from independent cohorts. This approach enabled a more comprehensive evaluation of the relationship between GDF15 expression and clinical parameters, thereby strengthening the overall interpretation of our results.

### 2.6. Statistical Analysis

**Figure 1:**
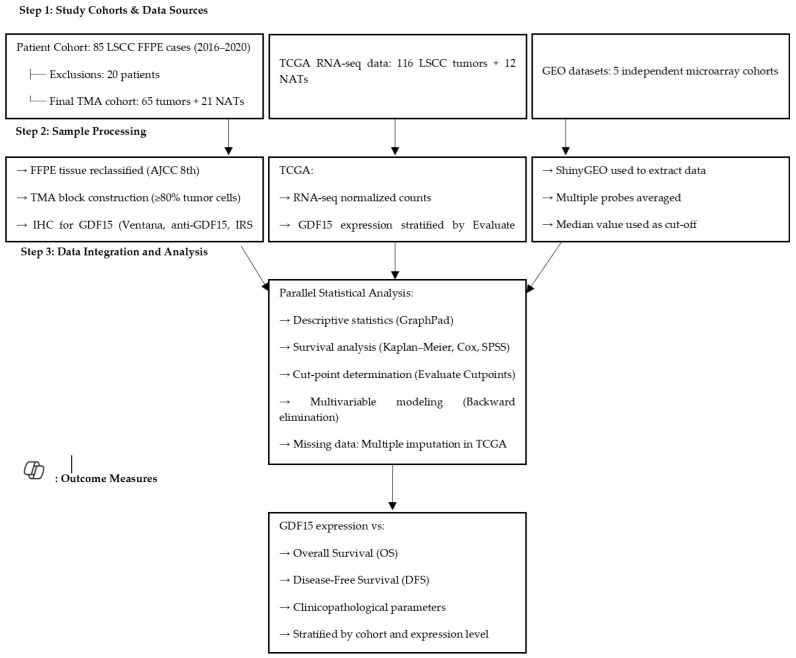
Schematic representation of the study design. The workflow integrates immunohistochemistry (IHC) data from the TMA cohort (*n* = 65 tumor samples, *n* = 21 normal adjacent tissues) with transcriptomic datasets from TCGA (RNA sequencing) and GEO (microarray). This multi-platform approach was used to evaluate the prognostic significance of GDF15 in laryngeal squamous cell carcinoma (LSCC), allowing for cross-cohort and cross-platform validation.

All statistical analyses were performed using GraphPad Prism (version 8.0, GraphPad Software, San Diego, CA, USA), SPSS (version 28.0, IBM Corporation, Armonk, NY, USA), or RStudio (version 1.3.1093) software packages. GraphPad Prism was used for descriptive statistics, normality testing (Shapiro–Wilk test), and group comparisons (Mann–Whitney U test and chi-squared test). SPSS was used for univariable and multivariable Cox proportional hazards regression analyses, including the backward elimination method and multiple imputation of missing data. Kaplan–Meier survival curves and log-rank tests were generated using SPSS. RStudio was used for additional data visualization and handling. The optimal expression thresholds for GDF15 were determined using the Evaluate Cutpoints software. Data distribution was determined using the Shapiro–Wilk test, and appropriate parametric or nonparametric statistical tests were applied. Overall survival (OS) was defined as the time from the date of curative surgery to death from any cause. Disease-free survival (DFS) was defined as the time from the date of curative surgery to the time of relapse or death. Patients who were alive at the last follow-up (11 January 2023) were censored. The median follow-up times, calculated using the reverse Kaplan–Meier method, were 49 months for OS and 46 months for DFS. The Kaplan–Meier method and log-rank test were used to depict and compare unadjusted survival curves for OS and DFS. Univariable and multivariable Cox proportional hazards regression analyses were conducted to explore the predictors of survival by estimating the hazard ratios (HR) and 95% confidence intervals (CI). A backward elimination procedure was used to build all multivariable models, with a significance level of *p* < 0.2 to enter the model and *p* ≤ 0.05 to stay. The proportional hazard assumption was verified by testing for significant interactions when each variable was entered as a time-based covariate. In the TCGA cohort, multiple imputation of missing variables for the Cox proportional hazards models was used to adjust for the bias of missing data; however, variables with amounts of missing data over 20% were not considered for the model. It was assumed that the missing data for the predictor variables occurred randomly. Combined estimates were obtained from 10 imputed datasets. To enhance clarity and underscore the methodological innovation of our study, we added a schematic overview (Figure 1) that illustrates the integrative design of our approach. This schematic outlines the parallel analysis of protein-level data (IHC from the TMA cohort) and transcriptomic-level data (RNA sequencing from TCGA and microarray from GEO), demonstrating how these independent sources complement each other to reinforce the validity and robustness of our findings. By integrating immunohistochemical profiling with large-scale public datasets, our design enabled cross-platform validation and highlighted the clinical relevance of GDF15 expression in LSCC from multiple analytical perspectives. Associations were considered statistically significant if the two-sided *p*-value was <0.05.

## 3. Results

### 3.1. Immunohistochemical Analysis of GDF15 Expression Comparing Tumor Tissue with Adjacent Normal Tissue

**Figure 2:**
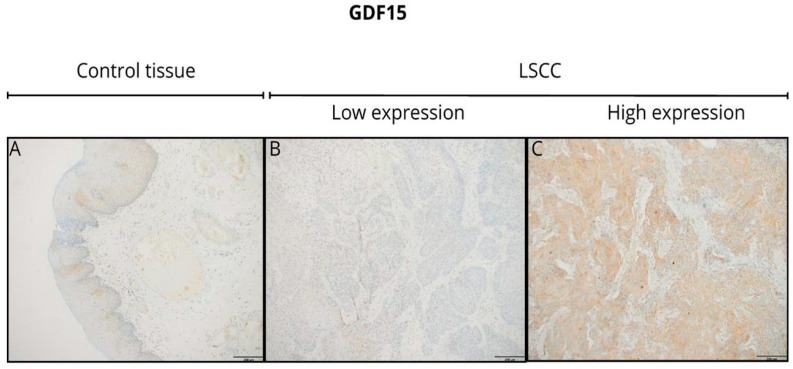
Representative images of immunohistochemical expression of GDF15 in laryngeal squamous cell carcinoma (LSCC) (**B**,**C**) and histologically normal tissue adjacent to the tumor (control tissue) (**A**). Original magnification: 10×. Scale bar = 200 µm.

At the protein level, GDF15 expression, assessed by immunohistochemistry (IHC), revealed primarily cytoplasmic staining at any level of expression intensity in tumor tissues and non-tumor tissues adjacent to the tumor. Representative IHC images demonstrated the variation in GDF15 staining intensity between the tumor tissues (Figure 2). However, tumor tissues showed a more heterogeneous and often stronger staining pattern than adjacent normal tissues. Additionally, the staining pattern observed in the tumor tissues exhibited significant heterogeneity, with variations in the intensity across different regions of the same sample. Although representative IHC images (Figure 2) demonstrated a stronger and more heterogeneous GDF15 staining pattern in tumor tissues than in adjacent normal tissues, the difference was not statistically significant. This discrepancy may be attributed to the relatively small number of high-quality NAT samples (*n* = 21), which limits the statistical power. Moreover, the semi-quantitative IRS scoring system used in this study, although standardized and widely applied, may not fully capture subtle differences in staining intensity and distribution across heterogeneous tumor regions. Finally, interindividual variability in baseline GDF15 expression among non-neoplastic tissues could further dilute the overall statistical differences between groups.

### 3.2. Comparison of GDF15 Expression Between Tumor Tissue and Non-Tumor Tissue in TMA and Publicly Available Cohorts

**Figure 3:**
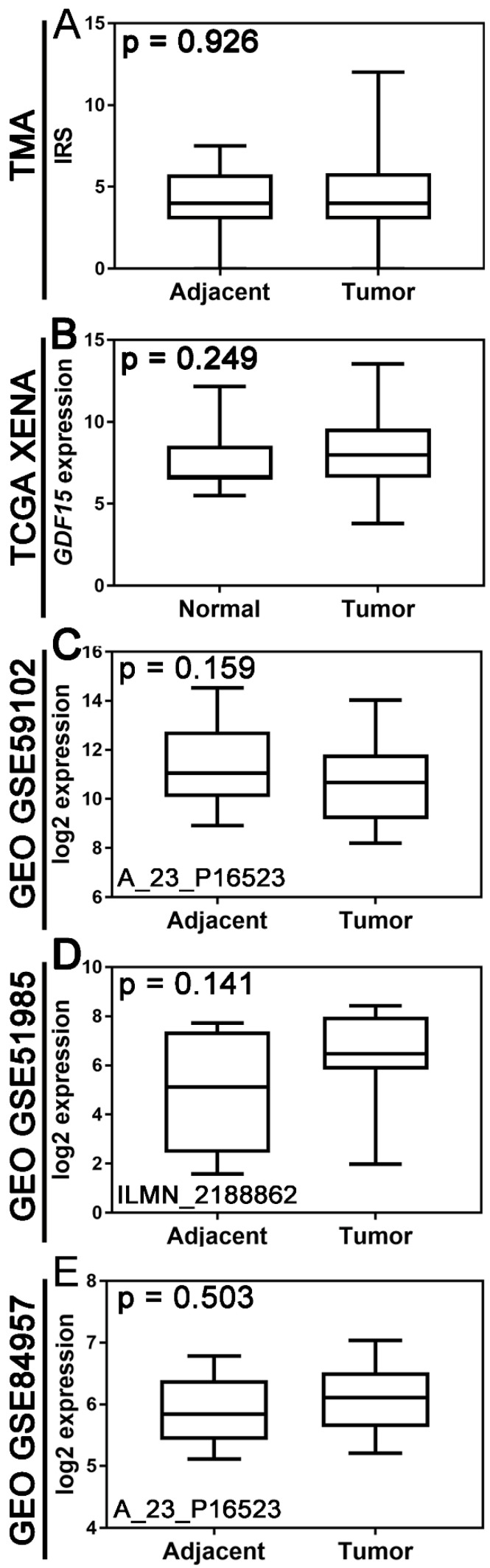
Expression of GDF15 in laryngeal squamous cell carcinoma compared to non-cancerous control tissue. Boxplot graphs of (**A**) GDF15 expression in tumor tissue (*n* = 65) and histologically normal tissue adjacent to the tumor (*n* = 21) of the institutional tissue microarray (TMA) cohort based on immunohistochemistry (IHC); (**B**) GDF15 expression levels in tumor tissue (*n* = 116) and normal solid tissue (*n* = 11) based on RNA-seq data from The Cancer Genome Atlas (TCGA) and Genotype-Tissue Expression (GTEx) databases, respectively, downloaded from the UCSC Xena Browser. (**C**) GDF15 expression in tumor tissue (*n* = 29) and histologically normal tissue adjacent to the tumor (*n* = 13) based on gene chip data derived from the Gene Expression Omnibus (GEO) database using the ShinyGEO web-based tool. The GSE number and probe information are provided. (**D**) GDF15 expression in tumor tissue (*n* = 10) and corresponding adjacent non-neoplastic tissue (*n* = 10) based on gene chip data derived from the Gene Expression Omnibus (GEO) database using the ShinyGEO web-based tool. The GSE number and probe information are provided. (**E**) GDF15 expression in tumor tissue (*n* = 9) and corresponding adjacent non-neoplastic tissue (*n* = 9) based on gene chip data derived from the Gene Expression Omnibus (GEO) database using the ShinyGEO web-based tool. The GSE number and probe information are provided.

There was no statistically significant difference in GDF15 expression between tumor and non-tumor tissues at either the protein or mRNA expression levels (Figure 3). Boxplot graphs in Figure 2 illustrate GDF15 expression levels in tumor tissue versus histologically normal tissue across various cohorts, including the institutional TMA, TCGA, and GEO datasets.

### 3.3. Stratified Analysis by Clinical and Lifestyle Variables

To assess the potential impact of key clinical and lifestyle factors on GDF15 expression, we performed subgroup analyses stratifying the data by sex, smoking status, and alcohol consumption status. These analyses did not reveal any statistically significant differences in GDF15 expression between subgroups (*p* > 0.05). Based on these findings, we combined the groups for further analyses. This result confirmed that the influence of these factors on GDF15 expression in our cohort was negligible.

### 3.4. Association Between GDF15 Expression and Clinicopathological Variables in TMA and TCGA Cohorts

After data dichotomization according to the established cut-off values, the TMA cohort included 28 (43.08%) samples with low and 37 (56.92%) samples with high GDF15 expression, and the TCGA cohort included 89 (76.72%) samples with low and 27 (23.28%) samples with high gene expression.

In the TMA LSCC cohort (Table 1), high GDF15 expression was significantly associated with less advanced pT stages (*p* = 0.02). Moreover, high GDF15 expression was significantly associated with a lower TNM stage (*p* = 0.019). A statistically significant relationship was also observed between GDF15 expression and the location of the primary tumor.

In the TCGA cohort, there were no statistically significant differences in clinicopathological variables depending on the GDF15 expression status (Table 2). However, the association between GDF15 expression and N status was of borderline statistical significance (*p* = 0.047; Table 2).

### 3.5. Relationships to Survival

**Figure 4:**
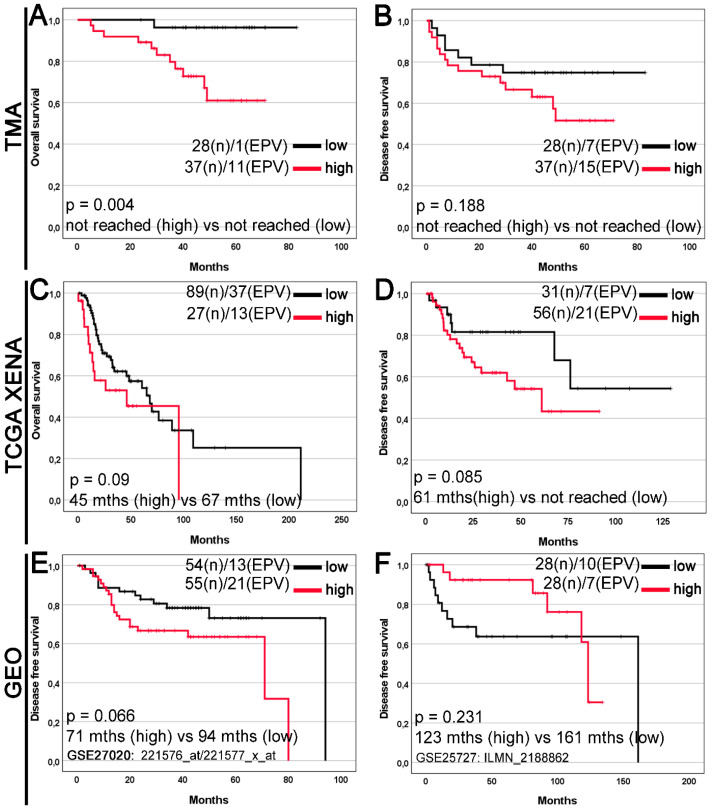
Kaplan–Meier curves for the overall survival (**A**,**C**) and disease-free survival (**B**,**D**–**F**) of laryngeal squamous cell carcinoma patients stratified by GDF15 expression. The survival curves were plotted based on the (**A**,**B**) immunohistochemistry data for the tissue microarray (TMA) cohort; gene expression levels for (**C**,**D**) The Cancer Genome Atlas (TCGA) dataset downloaded from the UCSC Xena Browser; (**E**,**F**) the Gene Expression Omnibus (GEO) datasets downloaded from the ShinyGEO web-based tool (the GSE number and probe information are shown). The cases were divided into two expression groups (low and high) according to (**A**–**D**) the optimal cut-off point determined by the Evaluate Cutpoints software and (**E**,**F**) the median expression. The results are shown with median OS or DFS (in months; abbreviated as mths) for high and low expression groups, as well as the *p*-value from the log-rank test. The number of cases (n) and events (EPV) in the low- and high-expression groups are displayed.

In Kaplan–Meier survival analysis of the TMA cohort, high GDF15 expression was significantly associated with reduced overall survival (OS) compared with low GDF15 expression (median OS: not reached vs. not reached; log-rank *p* = 0.004; Figure 4A), although there was no significant difference in disease-free survival (DFS) (median DFS: not reached vs. not reached; log-rank *p* = 0.188; Figure 4B). Furthermore, in the TCGA Xena and GEO cohorts, high GDF15 expression was not significantly associated with worse OS or DFS (Figure 4C–F). In the GEO cohort (Figure 4F), no significant association was observed between GDF15 expression and survival (*p* = 0.231).

**Figure 5:**
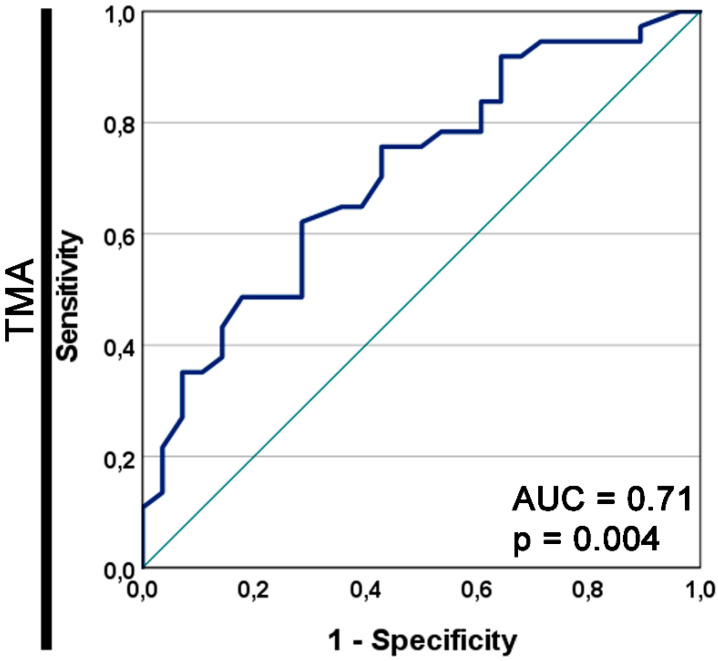
Receiver operating characteristic (ROC) curve for GDF15 as a prognostic marker for overall survival in the tissue microarray (TMA) cohort. The asymptotic *p*-value of the ROC curve is shown. AUC, area under the ROC curve.

The area under the prognostic ROC curve for GDF15 as a prognostic marker for OS in the TMA cohort was 0.71, with an asymptotic *p*-value of 0.004 (Figure 5).

As presented in Table 3, a univariate analysis of DFS in the TMA cohort indicated statistically significant univariable hazard ratios (HRs) for positive resection margins (HR 2.52, 95% CI 1.05–6.04, *p* = 0.038) and pT2 stage (HR 2.65, 95% CI 1.03–6.86, *p* = 0.044). In the multivariate Cox regression model, high GDF15 expression remained an independent predictor of disease-free survival (HR 2.98; 95% CI 1.16–7.65; *p* = 0.023). This suggests that elevated GDF15 levels may identify patients at an increased risk of recurrence, regardless of the tumor stage.

Additionally, TNM stage II (HR 4.64; 95% CI 1.53–14.13; *p* = 0.007) and stages III–IV (HR 3.13; 95% CI 1.06–9.27; *p* = 0.04) were also significantly associated with reduced DFS, confirming the expected clinical progression patterns. Notably, other variables included in the model were eliminated during backward selection due to a lack of statistical significance and were therefore excluded from the final model.

In the TCGA cohort, multivariate analysis revealed that sex (HR 0.30; 95% CI 0.15–0.59; *p* = 0.0006) and tumor grade G2 (HR 4.83; 95% CI 1.14–20.43; *p* = 0.032) were independent predictors of overall survival. Female patients and those with lower-grade tumors exhibited better prognoses, in line with established clinical expectations (Table 4).

The only independent predictor of disease-free survival was lymph node status. Patients with N1–N3 disease had significantly worse DFS (HR 2.69; 95% CI 1.14–6.35; *p* = 0.024), underscoring the prognostic importance of nodal involvement in LSCC.

## 4. Discussion

Growth differentiation factor 15 (GDF15) is a peptide hormone belonging to the transforming growth factor beta (TGFβ) superfamily that exhibits diverse physiological and pathological effects across multiple contexts. Under physiological conditions, GDF15 is present in various human tissues, including the placenta, liver, prostate, bladder, kidneys, colon, and endometrium, although in small quantities. Elevated serum concentrations of GDF15 have been associated with various malignancies, indicating its potential utility as a diagnostic biomarker for neoplastic diseases [7,25]. The pleiotropic effects of this protein have spurred extensive research into its role in the formation and progression of malignant tumors, as well as its involvement in cancer metastasis. Previous studies evaluating GDF15 as a marker for malignant tumors and metastasis diagnosis prompted us to investigate its expression in patients with laryngeal squamous cell carcinoma (LSCC). To our knowledge, this is the first study to evaluate GDF15 expression in LSCC, and our findings further support its potential as a prognostic biomarker.

In our study, we demonstrated that the lack of a statistically significant difference in GDF15 expression between tumor tissue and normal tissue adjacent to the tumor may suggest similar levels of activity of this protein in both types of tissue. However, it is important to consider the potential differences in cellular composition and microenvironment present in these two types of tissues, which could influence the interpretation of the results. The associations between GDF15 expression and clinical variables, such as T feature and TNM stage, exhibited variations between the tissue microarray (TMA) and The Cancer Genome Atlas (TCGA) cohorts, as indicated by the data dichotomization and analysis presented earlier. The TMA cohort assessed protein expression levels, whereas the TCGA and GEO cohorts measured molecular levels, that is, gene/mRNA expression levels. In the TMA cohort, consisting of 28 samples with low gene expression and 37 samples with high gene expression, the frequency of high and low GDF15 protein expression significantly differed in relation to the T feature according to the pT status and TNM stage. Notably, patients with stage I disease demonstrated a higher prevalence of high GDF15 protein expression than low expression levels. Conversely, no statistically significant differences in clinicopathological variables were observed in the TCGA cohort. The significant association between high GDF15 expression and poorer overall survival in patients with laryngeal squamous cell carcinoma, predominantly observed in the tissue microarray (TMA) dataset, may suggest the potential prognostic value of this protein. Third, methodological differences between the assessment of protein expression in the TMA cohort and mRNA expression in the TCGA and GEO datasets could also contribute to these discrepant findings. Finally, the relatively small sample size of our cohort may limit the statistical power, thereby affecting the interpretation of survival outcomes. However, the lack of significant differences in disease-free survival across all datasets necessitates further analysis, considering additional prognostic factors. The absence of a statistically significant difference in GDF15 expression between tumors and adjacent normal tissues does not negate its prognostic utility. This suggests that the prognostic relevance of GDF15 may stem from its functional role in the tumor microenvironment and systemic signaling, rather than its differential abundance alone. This pattern has also been observed for other biomarkers, where clinical outcomes are more tightly associated with systemic or functional effects than with absolute expression differences. The trends approaching statistical significance in DFS and overall survival observed in the TCGA and GEO datasets suggest the potential broader significance of GDF15 expression in cancer prognosis. Analysis of these trends indicates that higher levels of GDF15 may be associated with worse survival outcomes, which could have significant clinical implications. However, it is important to note that the significant association between high GDF15 expression and reduced overall survival was observed exclusively in the TMA cohort and not replicated in the TCGA or GEO datasets. This lack of validation across independent cohorts limits the generalizability of our findings and suggests that the prognostic value of GDF15 may be context-dependent or influenced by methodological factors, such as protein-level versus transcript-level assessment, cohort size, or clinical heterogeneity.

Moreover, the TMA cohort revealed a paradoxical pattern in which high GDF15 expression was associated with less advanced TNM and pT stages—typically linked to better prognosis—yet correlated with worse overall survival. This apparent contradiction may reflect the pleiotropic nature of GDF15, which not only marks local tumor behavior but may also indicate systemic effects, such as inflammation, cachexia, or immune modulation, that contribute to poorer outcomes independently of tumor stage.

These findings underscore the complex interplay of GDF15 in laryngeal squamous cell carcinoma and highlight the need for comprehensive investigations. Future studies with larger cohorts and robust event data are necessary to confirm these results, precisely delineate the prognostic role of GDF15, and explore its potential application in clinical practice.

Clinical studies have indicated its involvement in promoting metastasis [6,27]. Moreover, GDF15 expression is correlated with decreased survival in cancer patients, particularly in laryngeal squamous cell carcinoma, highlighting its potential prognostic value [6]. Additionally, the concentration of this marker has been shown to be significant in colorectal [28,29], gastric [30,31], pancreatic [32,33], breast [34,35], and prostate cancers [36,37]. However, to better understand the role of GDF15 as a prognostic biomarker, further research is needed, considering methodological limitations and other prognostic factors. Despite these limitations, our results suggest that GDF15 could be an important prognostic factor in various types of cancer, with significant clinical implications for cancer treatment and patient care.

The paradoxical observation that high GDF15 expression in the TMA cohort is associated with less advanced TNM and pT stages yet correlates with poorer overall survival, may also reflect the systemic effects of GDF15 that are not directly captured by local tumor characteristics. GDF15 acts as a stress-response cytokine and has been implicated in tumor-induced metabolic dysregulation, particularly anorexia and cachexia. Elevated circulating levels of GDF15 have been shown to contribute to weight loss, muscle wasting, and impaired tolerance to treatment, all of which may negatively affect overall survival, independent of tumor burden [38,39,40,41,42]. Thus, it is possible that increased GDF15 expression reflects an aggressive tumor phenotype associated with systemic catabolic effects, which could partly explain the discrepancy between the lower TNM stage and poor prognosis in our cohort.

This study had several limitations that must be acknowledged. First, the cohort size was very small, which reduced the statistical power and generalizability of our findings. Second, the low number of events per variable precluded us from performing Cox proportional hazards analysis in relation to overall survival time. These limitations underscore the need for caution when drawing definitive conclusions. Future studies with larger cohorts and more robust event data are necessary to validate our findings and provide more comprehensive insights into the role of GDF15 as a prognostic biomarker for laryngeal squamous cell carcinoma.

Our findings provide novel prognostic insights beyond the currently used LSCC biomarkers, such as EGFR, p16, and HPV status, which often lack consistency across cohorts. In contrast, GDF15 was independently associated with disease-free survival in multivariable models. This suggests that GDF15 may capture unique biological processes potentially related to tumor-induced systemic effects that are not reflected by traditional histopathological or molecular markers. Based on our findings, GDF15 shows potential as a prognostic biomarker in LSCC, as its elevated expression correlates with poor overall survival. Its integration into diagnostic panels could facilitate more accurate risk stratification and early detection of aggressive disease phenotypes when combined with other established markers. Furthermore, given its involvement in tumor progression and systemic effects, such as cachexia and metabolic dysregulation, targeting GDF15 or its downstream pathways may offer a novel therapeutic strategy. Future studies are warranted to evaluate the feasibility and clinical efficacy of incorporating GDF15 into routine diagnostic workflows and as a potential target for therapeutic intervention in LSCC.

We refined our study parameters, rationale, and methods to enhance the clarity and robustness of our findings. Detailed justifications for the sample selection, data analysis, and clinical relevance of GDF15 are provided throughout the manuscript, thereby strengthening the validity of our results and the overall solidity of the study.

## 5. Conclusions

The study results showed no significant difference in GDF15 expression between the tumor and adjacent normal tissues. However, variability in GDF15 expression was observed in relation to clinical variables exclusively in the TMA cohort. Although elevated GDF15 levels were significantly associated with poorer overall survival in the TMA cohort, this finding was not consistently replicated in the TCGA and GEO datasets. Therefore, although GDF15 may have prognostic potential, its utility as a robust biomarker requires further validation in larger, independent, and clinically diverse populations.

## Figures and Tables

**Table 1:** Association of GDF15 with clinicopathological variables in the TMA cohort.

Variable	*n* (%)	GDF15 Low	GDF15 High	*p*-Value
**Demographic variables**				
Age (years) ≤ 60	17 (26.15)	7 (41.18)	10 (58.82)	>0.999
Age (years) > 60	48 (73.85)	21 (43.75)	27 (56.25)	>0.999
Gender: Male	55 (84.62)	22 (40.00)	33 (60.00)	0.306
Gender: Female	10 (15.38)	6 (60.00)	4 (40.00)	0.306
**Tumor characteristics**				
Tumor grade G1	16 (24.62)	6 (37.50)	10 (62.50)	0.854
Tumor grade G2	45 (69.23)	20 (44.44)	25 (55.56)	0.854
Tumor grade G3	4 (6.15)	2 (50.00)	2 (50.00)	0.854
pT status T1	32 (49.23)	8 (25.00)	24 (75.00)	0.020
pT status T2	23 (35.39)	14 (60.87)	9 (39.13)	0.020
pT status T3–T4	10 (15.38)	6 (60.00)	4 (40.00)	0.020
pN status N0	52 (80.00)	20 (38.46)	32 (61.54)	0.210
pN status N1–N2	13 (20.00)	8 (61.54)	5 (38.46)	0.210
Resection margin: negative	50 (76.92)	23 (46.00)	27 (54.00)	0.554
Resection margin: positive	15 (23.08)	5 (33.33)	10 (66.67)	

**Table 2:** Association of GDF15 with clinicopathological variables in the TCGA cohort.

Variable	*n* (%)	GDF15 Low	GDF15 High	*p*-Value
**Demographic variables**				
Age < 60	40 (34.48)	33 (82.50)	7 (17.50)	0.358
Age ≥ 60	76 (65.52)	56 (73.68)	20 (26.32)	
Male	96 (82.76)	74 (77.08)	22 (22.92)	0.780
Female	20 (17.24)	15 (75.00)	5 (25.00)	
**Tumor grade**				
G1	8 (6.90)	7 (87.50)	1 (12.50)	0.111
G2	71 (61.21)	50 (70.42)	21 (29.58)	
G3–G4	33 (28.45)	29 (87.88)	4 (12.12)	
N/A	4 (3.45)	–	–	
**T status**				
T1	7 (6.03)	5 (71.43)	2 (28.57)	0.966
T2	20 (17.24)	15 (75.00)	5 (25.00)	
T3	33 (28.45)	25 (75.76)	8 (24.24)	
T4	56 (48.28)	44 (78.57)	12 (21.43)	
**N status**				
N0	52 (44.83)	35 (67.31)	17 (32.69)	**0.047**
N1–N2	63 (54.31)	53 (84.13)	10 (15.87)	
N/A	1 (0.86)	–	–	
**TNM stage**				
I–II	15 (12.93)	10 (66.67)	5 (33.33)	0.327
III	19 (16.38)	13 (68.42)	6 (31.58)	
IV	82 (70.69)	66 (80.49)	16 (19.51)	
**Lymphovascular invasion**				
No	42 (36.21)	33 (78.57)	9 (21.43)	>0.999
Yes	36 (31.03)	28 (77.78)	8 (22.22)	
N/A	38 (32.76)	–	–	
**Alcohol consumption**				
No	39 (33.62)	33 (84.62)	6 (15.38)	0.240
Yes	75 (64.66)	55 (73.33)	20 (26.67)	
N/A	2 (1.72)	–	–	

**Table 3:** Univariable and Multivariable Cox Regression Analyses for Disease-Free Survival in TMA Cohort.

Variable	*n*/EPV	Univariable HR	95% CI	*p*	Multivariable HR	95% CI	*p*
			**Lower–Upper**			**Lower–Upper**	
**GDF15 (high vs. low)**	37/15–28/7	1.81	0.74–4.43	0.197	**2.98**	**1.16–7.65**	**0.023**
**TNM stage**							
I (reference)	30/6	REF.	–	–	REF.	–	–
II vs. I	17/8	2.92	1.01–8.43	0.048	**4.64**	**1.53–14.13**	**0.007**
III–IV vs. I	18/8	2.23	0.77–6.44	0.138	**3.13**	**1.06–9.27**	**0.040**

**Abbreviations**: HR—hazard ratio; CI—confidence interval; EPV—events per variable; REF.—reference category; GDF15—growth differentiation factor 15; TNM—tumor-node-metastasis.

**Table 4:** Univariable and multivariable Cox analyses of overall survival (*n* = 116) and disease-free survival (*n* = 87) in the TCGA cohort.

Outcome	Variable	Multivariable HR	95% CI	*p*
Overall Survival				
Sex (male vs. female)		0.30	0.15–0.59	0.0006
Tumor grade (G2 vs. G1)		4.83	1.14–20.43	0.032
	G3–G4 vs. G1	2.13	0.47–9.70	0.326
Disease-Free Survival				
N stage (N1–N3 vs. N0)		2.69	1.14–6.35	0.024

**Abbreviations**: HR—hazard ratio; CI—confidence interval.

## Data Availability

Data supporting the reported results are available from the corresponding author upon reasonable request.

## Abbreviations

The following abbreviations are used in this manuscript:

AJCC	American Joint Committee on Cancer
DFS	Disease-Free Survival
FFPE	Formalin-Fixed, Paraffin-Embedded
GDF15	Growth Differentiation Factor 15
GEO	Gene Expression Omnibus
GTEx	Genotype-Tissue Expression
HR	Hazard Ratio
IHC	Immunohistochemistry
IRS	Immunoreactive Score
LSCC	Laryngeal Squamous Cell Carcinoma
N/A	Not Available
OS	Overall Survival
ROC	Receiver Operating Characteristic
TCGA	The Cancer Genome Atlas
TGF-β	Transforming Growth Factor Beta
TMA	Tissue Macroarray
